# Usefulness and Limitations of Cryopreservation for Immunocytochemical Staining of Canine Cytological Specimens for Detection of Cytokeratin and Vimentin

**DOI:** 10.3390/vetsci10020153

**Published:** 2023-02-14

**Authors:** Yu Furusawa, Mariko Shima-Sawa, Tatsuro Hifumi, Noriaki Miyoshi, Osamu Yamato, Akira Yabuki

**Affiliations:** 1 Kagoshima University Veterinary Teaching Hospital, Joint Faculty of Veterinary Medicine, Kagoshima University, 1-21-24 Korimoto, Kagoshima 890-0065, Japan; 2 Laboratory of Veterinary Clinical Pathology, Joint Faculty of Veterinary Medicine, Kagoshima University, 1-21-24 Korimoto, Kagoshima 890-0065, Japan; 3 Laboratory of Veterinary Histopathology, Joint Faculty of Veterinary Medicine, Kagoshima University, 1-21-24 Korimoto, Kagoshima 890-0065, Japan

**Keywords:** cryopreservation, cytokeratin, diagnostic cytology, dog, immunocytochemistry, smear, vimentin

## Abstract

**Simple Summary:**

Immunocytochemistry is a useful diagnostic tool in cytology; nonetheless, compared with pathological tissue specimens, cytological specimens have a crucial issue with long-term storage of antigenicity. A convenient and effective method is required for the storage of air-dried smears, which are commonly used for cytology, especially in small animal medicine. In the present study, we evaluated the usefulness of cryopreservation of air-dried smears for immunocytochemical staining for cytokeratin and vimentin, which can help distinguish between epithelial and non-epithelial tumors. The study results revealed that immunoreactivity for cytokeratin and vimentin could be detected for at least 33 months in unfixed smear samples stored in a freezer, and demonstrated the usefulness and effectiveness of cryopreserved air-dried smears in veterinary immunocytochemistry.

**Abstract:**

Immunocytochemistry is an advanced diagnostic tool for identifying the origin of tumor cells. This study aimed to highlight the usefulness of cryopreserved, air-dried cytological samples in detecting cytokeratin and vimentin. Air-dried cytological smear samples were prepared from a total of 39 resected canine tumors and stored in a medical freezer without fixation. The duration of cryopreservation ranged from 2 to 56 months. The same tumors were processed for routine histopathological examination. Based on the morphological diagnosis, cryopreserved FNA smears from epithelial tumors were stained by enzymatic immunocytochemistry (ICC) for cytokeratin; those from mesenchymal and melanocytic tumors were stained by ICC for vimentin. To ascertain the positivity of tumor cells to the selected markers, tissue paraffin-embedded sections were also stained by immunohistochemistry (IHC) for the same markers. Immunoreactivity for cytokeratin was detected in cryopreserved cytological smears for a maximum of 46 months. Immunoreactivity for vimentin was clearly detected for 33 months. Smears stored at room temperature for 1 week did not show any signals under immunocytochemical examination. Thus, immunocytochemistry for cytokeratin and vimentin can be safely applied to air-dried smears cryopreserved in a freezer for at least 33 months.

## 1. Introduction

Immunocytochemistry (ICC) is an advanced technique that can aid in identifying the origin of malignant cells in carcinomas, sarcomas, and lymphomas [1,2,3,4,5]. ICC is usually performed in cases in which the tumor’s origin is difficult to distinguish using conventional cytological methods involving Romanowsky stains. In veterinary clinicopathological laboratories, non-stained smears are often submitted from clinicians for ICC after standard diagnosis using Romanowsky stains. However, air-dried cytological smears stored at room temperature (RT) have poor antigen preservation, and immediate staining after sample preparation has been recommended for successful ICC [6,7]. Therefore, a simple method for preserving samples is required, particularly in veterinary clinics without laboratories or clinicians with expertise in ICC.

Cryopreservation is the most common method used for the long-term storage of paraffin-embedded and frozen sections for immunohistochemistry (IHC) [8,9]. For ICC, this method has been applied in human clinicopathological laboratories [10], and it is also applied empirically in several clinicopathological laboratories. If ICC can be utilized using long-stored smear samples, such samples can be used to verify the accuracy of the initial diagnosis, especially in patients with an unexpected prognosis and clinical behaviors. Furthermore, it can serve as a useful tool in retrospective or prospective studies, thereby contributing to the exploration of new and effective markers for predicting and evaluating the clinical course of neoplastic diseases in dogs and cats. Nonetheless, the reliability of ICC with freezer-stored, unfixed, air-dried smears have not been previously evaluated for veterinary specimens. Among the various diagnostic markers that can be used in dogs and cats, cytokeratin and vimentin are representative markers for differentiating the origins between epithelial and non-epithelial cells [1,2,3,5]. Additionally, the protocol of ICC using air-dried smears for detecting these markers has been established [3,5].

If ICC can be utilized using long-stored smear samples, such samples can be used to re-evaluate diagnoses, especially in patients with an unexpected prognosis and clinical behaviors. Furthermore, it can serve as a useful tool in retrospective or prospective studies, thereby contributing to the exploration of new and effective markers for predicting and evaluating the clinical course of neoplastic diseases in dogs and cats. With this background, the present study was designed to evaluate the efficacy of cryopreservation of canine cytology samples by utilizing freezer-stored unfixed smears. 

## 2. Materials and Methods

### 2.1. Samples

The experiments in the present study were conducted in accordance with the Guidelines for Veterinary Clinical Research of Kagoshima University in Japan (approval No. KVH190001). All 39 samples used in the present study were prepared from surgical excisional biopsies obtained from dogs that were admitted to Kagoshima University Veterinary Teaching Hospital, Japan. All cytological smears were prepared using fine-needle aspiration (FNA) or impression before formalin fixation for routine histopathological examination. After the smears had completely air-dried, some of them were immediately stained with standard Giemsa stain (May–Grünwald–Giemsa). However, the remaining ones were stored unfixed in a medical freezer (−30 °C) within 24 h after sampling. The tumor specimens from dogs were fixed in 10% neutral buffered formalin and subjected to histopathological analysis by veterinary pathologists (T.H. and N.M.).

The following criteria were used for case selection: (1) a clear histopathological diagnosis of epithelial, mesenchymal or melanocytic tumors; (2) a cytological diagnosis corresponding to the histopathological diagnosis; and (3) a large number of tumor cells with adequate cytomorphology on the smear slides. On the basis of these criteria, samples from 37 dogs were selected for the study. The duration of storage of these selected samples varied from 2 to 56 months.

The median age of the cases was 10.9 years (range: 4–15 years). All the dogs were neutered, and over half of those were males (57.1%). The breeds of dogs were as follows: Miniature Dachshund (*n* = 6), mongrel (*n* = 5), Labrador Retriever (*n* = 4), Shih Tzu (*n* = 3), French Bulldog (*n* = 3), Chihuahua (*n* = 3), Shetland Sheepdog (*n* = 3), Yorkshire Terrier (*n* = 1), Papillon (*n* = 1), Pembroke Welsh Corgi (*n* = 1), West Highland White Terrier (*n* = 1), Airedale Terrier (*n* = 1), Shiba Inu (*n* = 1), American Cocker Spaniel (*n* = 1), Border Collie (*n* = 1), Great Pyrenees (*n* = 1), Pomeranian (*n* = 1), Miniature Schnauzer (*n* = 1), and Golden Retriever (*n* = 1).

Smears from two cases (adenoma and gastrointestinal stromal tumor) were intentionally stored at room temperature (RT) for one week in a non-air-conditioned room to evaluate the suitability of room temperature-stored smears for ICC. 

### 2.2. Detection of Cytokeratin and Vimentin

The primary antibodies were mouse monoclonal anti-cytokeratin (clone AE1/AE3, ready-to-use solution, Dako, Glostrup, Germany), mouse monoclonal anti-human vimentin (clone V9, 1:100 dilution; Thermo Fisher Scientific, Waltham, MA, USA), and rabbit monoclonal anti-human vimentin (clone SP20, 1:200 dilution; Spring Bioscience, Pleasanton, CA, USA). The cross-reactivity of these antibodies for canine tissues has been demonstrated in previous reports [3,5,11,12]. Normal mouse IgG (Dako) and normal rabbit IgG (Dako) were used as the negative control smears. The secondary antibodies were biotin-labeled horse anti-mouse IgG (1:200 dilution; Vector Laboratories, Burlingame, CA, USA) and biotin-labeled horse anti-rabbit IgG (1:200 dilution; Vector Laboratories, Burlingame, CA, USA). Moreover, 0.25% casein/10 mM phosphate-buffered saline (PBS) was used as the blocking solution and dilutant of the antibodies. The other reagents were ready-to-use peroxidase-labeled streptavidin (KPL, Gaithersburg, MD, USA; Vector Laboratories) and 3,3′-diaminobenzidine (DAB) chromogen (DAB-buffer tablet; Merck, Darmstadt, Germany). 

ICC was performed following a previously described method [5]. Briefly, the smear slides were immediately and completely dried using a hair dryer on the cold air setting after removing them from the freezer. Following fixation with cold acetone for 1 min, the slides were washed with PBS for 10 s, blocked for 10 min at RT, and incubated with the primary antibody for 10 min at 37 °C. After thorough washing with PBS, the slides were incubated with the secondary antibody for 10 min at 37 °C and washed with PBS. Thereafter, they were incubated using peroxidase-conjugated streptavidin for 10 min at 37 °C. After washing with PBS, a DAB solution was applied for 5 min to detect the immunoreactivity. The reaction was stopped with cold distilled water. Counterstaining was performed with Carrazi’s hematoxylin. Finally, the specimens were air-dried, penetrated with xylene, and mounted with a coverslip.

Sections 3 μm thick were prepared from formalin-fixed paraffin-embedded tissues and applied for IHC. The reagents for IHC were the same as for those for ICC. The staining method was similar to that conducted for ICC; however, the primary antibodies were incubated for 20 min at room temperature, the secondary antibody was incubated for 30 min at room temperature, and the peroxidase-conjugated streptavidin was incubated for 30 min.

All smears and sections were evaluated as positive (+) or negative (−) for immunoreactivity using light microscopy. The signal strengths were compared between ICC samples and negative controls. The ICC signals were also compared with standard IHC signals.

## 3. Results

In 37 cases in which freezer-stored smears were analyzed in the present study, 20, 11, and 6 cases were histopathologically diagnosed as epithelial, mesenchymal, and melanocytic tumors, respectively. The cytokeratin-positive immunoreactivity in the tumor cells was demonstrated in all the epithelial tumor cases by IHC, and the vimentin-positive immunoreactivity in the tumor cells was also demonstrated in the mesenchymal tumor and melanocytic tumor cases by IHC (Table 1).

**Figure 1:**
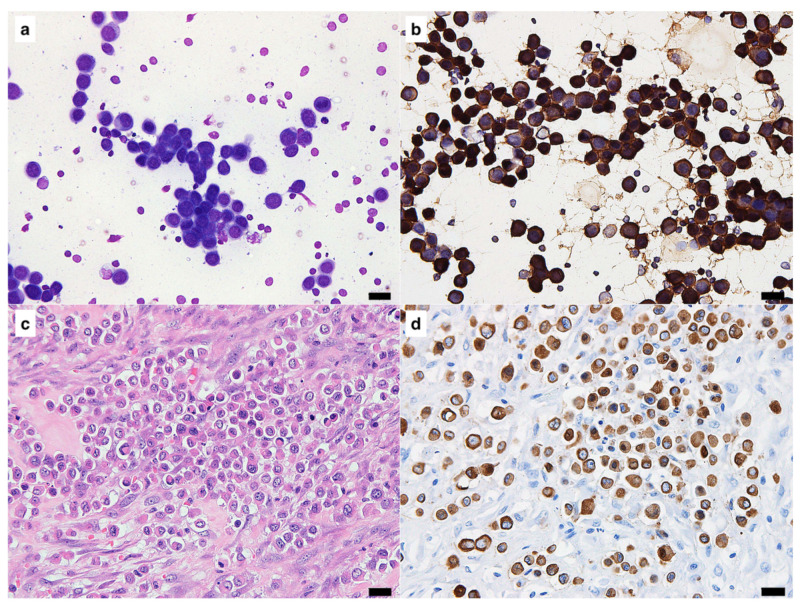
A sample diagnosed as undifferentiated carcinoma of the mammary gland was stored at −30 °C for 46 months. (**a**) Standard Giemsa stain. (**b**) Detection of cytokeratin by immunocytochemistry (ICC). (**c**) Standard hematoxylin and eosin stain. (**d**) Detection of cytokeratin detection by immunohistochemistry (IHC). Positive signals for cytokeratin in the tumor cells were observed in both the ICC and IHC samples. In the ICC and IHC panels: 3,3′-diaminobenzidine (DAB) chromogen, hematoxylin counterstain. Scale bars = 20 μm.

**Figure 2:**
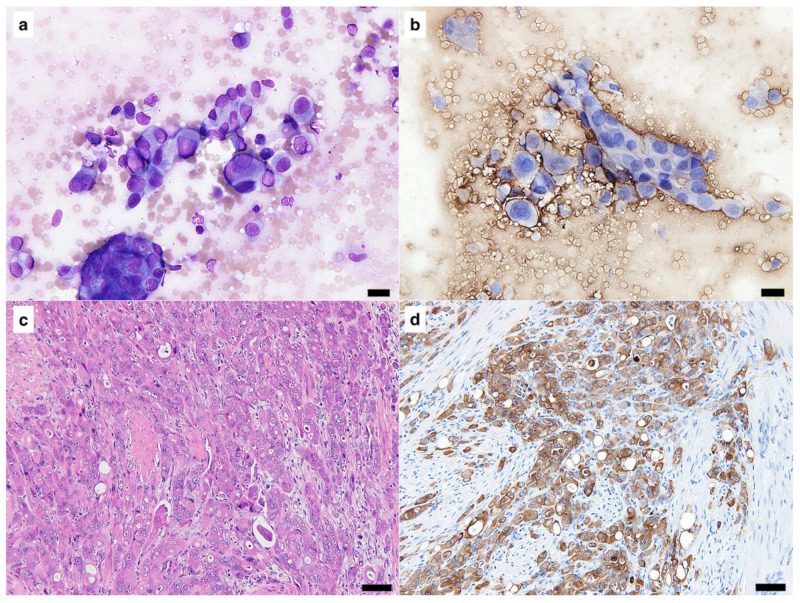
A sample diagnosed as transitional cell carcinoma of the urethra was stored at −30 °C for 56 months. (**a**) Standard Giemsa stain. (**b**) Detection of cytokeratin by immunocytochemistry (ICC). (**c**) Standard hematoxylin and eosin stain. (**d**) Detection of cytokeratin by immunohistochemistry (IHC). Positive signals for cytokeratin in the tumor cells were observed in samples stained using IHC but not in those stained using ICC. In the ICC and IHC panels: 3,3′-diaminobenzidine (DAB) chromogen, hematoxylin counterstain. Scale bars = 20 μm.

**Figure 3:**
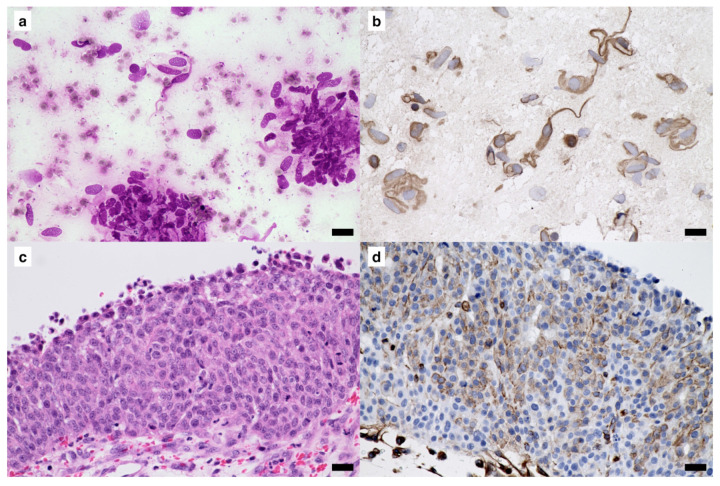
A sample diagnosed as gastrointestinal stromal tumor of the jejunum was stored at −30 °C for 33 months. (**a**) Standard Giemsa stain. (**b**) Detection of vimentin by immunocytochemistry (ICC). (**c**) Standard hematoxylin and eosin stain. (**d**) Detection of vimentin detection by immunohistochemistry (IHC). Positive signals for vimentin in the tumor cells were observed in samples stained using IHC and ICC. In the ICC and IHC panels: 3,3′-diaminobenzidine (DAB) chromogen, hematoxylin counterstain. Scale bars = 20 μm.

In ICC using freezer-stored smears, specific immunoreactivity was observed in 35 out of 37 (94.6%) cases (Table 1). Immunoreactivity for cytokeratin was clearly detected in 18 of 20 (90%) cases of epithelial tumors. The two cases cryopreserved for 56 months were both negative for cytokeratin with ICC. Immunoreactivity for vimentin was safely detected with ICC in all 11 mesenchymal and 6 melanocytic tumor cases. Figure 1 shows the findings of a case which was cryopreserved for 46 months. This case was diagnosed as undifferentiated carcinoma of the mammary gland, and clear immunoreactivity for cytokeratin was obtained by ICC (Figure 1b,d). Figure 2 shows the findings of a case, for which the smear was cryopreserved for 56 months. This case was diagnosed as transitional cell carcinoma of the urethra, and ICC was negative for cytokeratin (Figure 2b), although positive immunoreactivity was demonstrated with IHC (Figure 2d). Figure 3 shows the case of a smear cryopreserved for 33 months. This case was diagnosed as gastrointestinal stromal tumor of the jejunum, and clear immunoreactivity for vimentin with good-quality visualization was obtained not only by IHC but also by ICC (Figure 3b,d).

In cases of smears intentionally left at room temperature for 1 week in a non-air-conditioned room, no immunoreactivity was detected by ICC. In a case of mammary gland adenoma, the immunoreactivity for cytokeratin was not detected by ICC but was detected by IHC. In a case of gastrointestinal stromal tumor, the immunoreactivity for vimentin was also not detected by ICC but was detected by IHC.

## 4. Discussion

In the present study, stable immunoreactivity for cytokeratin and vimentin was safely detected in smears that were cryopreserved for an extremely long time. Cytokeratin could be detected clearly even in a sample cryopreserved for 46 months, and vimentin could be detected in samples cryopreserved for 33 months. Although we were unable to test samples cryopreserved for over 33 months, more extended storage could be applicable for the detection of vimentin. However, the present findings indicate that the antigenicity would not be permanently preserved if the samples were stored in a medical freezer similar to that used in the present study, because two samples from epithelial tumors cryopreserved for 56 months showed no specific signals for cytokeratin.

Whether the cytological smears should be cryopreserved before or after fixation is an important consideration. In a previous report, cryopreservation of air-dried smears after formalin-fixation was suspected to provide long-term antigen stability. However, in that previous study [13], antigen retrieval by heating of the slides with a citrate buffer was required to detect the immunoreactivity by ICC. This method may lead to detachment of the cells from the slide, and this error sometimes occurs if ICC is carried out with antigen retrieval by heating. Moreover, formalin is not always universal for ICC with air-dried smears. Although it is suitable for the detection of CD antigens for lymphocyte subtyping [4], it is unsuitable for the detection of cytokeratin and vimentin [5]. In our previous study, acetone, 10% neutral buffered formalin and 95% ethanol were tested as a fixative for detection of cytokeratin, vimentin, and S-100 protein by ICC with air-dried smears prepared from tumors of dogs. We confirmed that acetone provided sufficiently strong signals without antigen retrieval [5]. In the present study, the ICC method was based on the one in our previous study [5]. We observed that immunoreactivity for cytokeratin and vimentin remained stable over a long period of time, even though the air-dried smears were directly cryopreserved without fixation. This simplicity of cryopreserving smear samples would be a strength not only for general clinics but also clinicopathological laboratories.

The smears left at RT for 1 week were also tested in the present study, and immunoreactivity for both cytokeratin and vimentin was not detected in these samples. On the contrary, air-dried smears stored at RT for a longer period (20 weeks) could reportedly be used for ICC without the loss of antigenicity [2]; thus, 1 week of RT storage may seem too short for losing the antigenicity of the smear samples. However, there is a possible explanation for the loss of antigenicity in such a short time. The present study was conducted during the summer in Japan, when temperatures and humidity are high. Furthermore, our facility is located in an area that is even hotter and more humid than most areas. We intentionally left the RT samples in a non-air-conditioned room in the present test, and our results demonstrated that such a harsh storage environment could result in early loss of antigenicity through temperature and humidity. Thus, we recommend that the remaining smears should be cryopreserved, even if only for a short time, since storage at room temperature does not allow for standardization of storage conditions.

Cryopreservation of smear specimens is a simple and convenient method that can be performed without any special equipment. Although the present analysis was performed for cytokeratin and vimentin, ICC using cryopreserved smears would possibly be applicable to the detection of other antigens. Applicable ICC for the samples with long-term preservation possesses the strength to perform retrospective or prospective analyses using stored smear samples. In addition, if a re-evaluation of the diagnoses of the cases is required, cryopreserved smear samples would be good materials for ICC. Nevertheless, this study has some limitations. We did not evaluate the specimens stored at RT in a temperature-controlled environment, refrigerator, or home freezer. The period for cryopreservation could not be matched between evaluations of cytokeratin and evaluations of vimentin because this study was performed by utilizing freezer-stored samples that were not required after diagnosis. Other markers such as CD antigens were not examined. Therefore, further studies are required to determine the efficacy of the cryopreservation of samples for ICC in veterinary cytology.

## 5. Conclusions

The present study evaluated the efficacy of the cryopreservation of unfixed, air-dried smears for ICC in the detection of cytokeratin and vimentin. Freezer-stored smear samples from canine epithelial and mesenchymal tumor tissues were selected and tested. The duration of the storage of samples varied from 2 to 56 months. Thus, ICC for cytokeratin and vimentin can be safely used for unfixed and air-dried smears preserved in a freezer for at least 33 months.

## Figures and Tables

**Table 1:** Storage period and detection of cytokeratin or vimentin by ICC in canine neoplastic tissues.

Storage Period, Temperature	Sites	Histopathological Diagnosis	Primary Antibody, Clone	Signal	
				IHC	ICC
56 months, −30 °C	Mammary gland	Adenosquamous carcinoma	Cytokeratin, AE1/AE3	+	−
56 months, −30 °C	Urethra	Transitional cell carcinoma	Cytokeratin, AE1/AE3	+	−
46 months, −30 °C	Mammary gland	Undifferentiated carcinoma	Cytokeratin, AE1/AE3	+	+
34 months, −30 °C	Mammary gland	Complex adenoma	Cytokeratin, AE1/AE3	+	+
30 months, −30 °C	Perianal	Perianal gland adenoma	Cytokeratin, AE1/AE3	+	+
30 months, −30 °C	Rectum	Adenocarcinoma	Cytokeratin, AE1/AE3	+	+
30 months, −30 °C	Skin (trunk)	Trichoepithelioma	Cytokeratin, AE1/AE3	+	+
29 months, −30 °C	Mammary gland	Tubulopapillary carcinoma	Cytokeratin, AE1/AE3	+	+
28 months, −30 °C	Skin (forehead)	Sebaceous adenoma	Cytokeratin, AE1/AE3	+	+
27 months, −30 °C	Perianal	Perianal gland adenoma	Cytokeratin, AE1/AE3	+	+
26 months, −30 °C	Mammary gland	Lipid-rich carcinoma	Cytokeratin, AE1/AE3	+	+
25 months, −30 °C	Mammary gland	Mammary adenoma	Cytokeratin, AE1/AE3	+	+
24 months, −30 °C	Kidney	Renal carcinoma	Cytokeratin, AE1/AE3	+	+
20 months, −30 °C	Thyroid gland	Thyroid adenoma	Cytokeratin, AE1/AE3	+	+
15 months, −30 °C	Rectum	Adenocarcinoma	Cytokeratin, AE1/AE3	+	+
15 months, −30 °C	Salivary gland	Salivary adenocarcinoma	Cytokeratin, AE1/AE3	+	+
12 months, −30 °C	Lung	Squamous cell carcinoma	Cytokeratin, AE1/AE3	+	+
9 months, −30 °C	Intraperitoneum	Islet cell carcinoma	Cytokeratin, AE1/AE3	+	+
7 months, −30 °C	Lung	Adenocarcinoma	Cytokeratin, AE1/AE3	+	+
3 months, −30 °C	Liver	Hepatocellular adenoma	Cytokeratin, AE1/AE3	+	+
1 week, RT	Mammary gland	Adenoma	Cytokeratin, AE1/AE3	+	−
33 months, −30 °C	Jejunum	Gastrointestinal stromal tumor	Vimentin, SP20	+	+
31 months, −30 °C	Mandibular	Melanoma	Vimentin, SP20	+	+
29 months, −30 °C	Mediastinum	Osteosarcoma	Vimentin, SP20	+	+
26 months, −30 °C	Jejunum	Gastrointestinal stromal tumor	Vimentin, SP20	+	+
26 months, −30 °C	Hindlimb	Malignant peripheral nerve sheath tumor	Vimentin, SP20	+	+
24 months, −30 °C	Spleen	Malignant stromal tumor	Vimentin, V9	+	+
24 months, −30 °C	Mandible	Melanoma	Vimentin, SP20	+	+
23 months, −30 °C	Mandible	Melanoma	Vimentin, V9	+	+
23 months, −30 °C	Skin (jugular)	Malignant peripheral nerve sheath tumor	Vimentin, SP20	+	+
20 months, −30 °C	Skin (forelimb)	Malignant peripheral nerve sheath tumor	Vimentin, V9	+	+
18 months, −30 °C	Abdominal wall	Liposarcoma	Vimentin, SP20	+	+
18 months, −30 °C	Mandible	Melanoma	Vimentin, SP20	+	+
17 months, −30 °C	Liver	Angiosarcoma	Vimentin/V9	+	+
17 months, −30 °C	Abdominal wall	Liposarcoma	Vimentin, SP20	+	+
15 months, −30 °C	Mandible	Melanoma	Vimentin, SP20	+	+
14 months, −30 °C	Mandible	Melanoma	Vimentin, SP20	+	+
2 months, −30 °C	Testis	Interstitial cell tumor	Vimentin, V9	+	+
1 week, RT	Small intestine	Gastrointestinal stromal tumor	Vimentin, SP20	+	−

IHC, immunohistochemistry; ICC, immunocytochemistry; RT, room temperature; +, positive immunoreactivity; −, negative immunoreactivity.

## Data Availability

The data presented in this study are available on request from the corresponding author.
